# Supplementary material for: Mangiferin-Loaded Smart Gels for HSV-1 Treatment
Source: Pharmaceutics. 2021 Aug 24;13(9):1323. doi: 10.3390/pharmaceutics13091323 (PMC8465222; doi:10.3390/pharmaceutics13091323)
Supplement: Supplementary file 1 [file pharmaceutics-13-01323-s001.zip › pharmaceutics-1332347-supplementary.pdf]

pharmaceutics

Supplementary Material: Mangiferin-loaded smart gels for HSV-1 treatment

Mariaconcetta Sicurella, Maddalena Sguizzato, Rita Cortesi, Nicolas Huang, Fanny Simelière, Leda Montesi, Peggy Marconi and Elisabetta Esposito

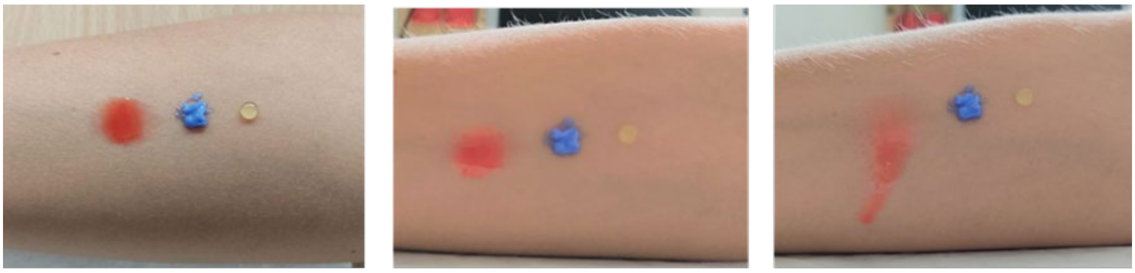

(a) (b) (c)

Figure S1. Comparative in vivo skin adhesion test performed after the application of LO, PLO-3, and PG, colored in red, blue, and yellow, respectively. The images were taken on a forearm lying horizontally (**a**) immediately after gel application and on a forearm in the vertical position 1 (**b**) or 10 (**c**) min after gel application.
